# Morphogenesis of Bullet-Shaped Rabies Virus Particles Regulated by TSG101

**DOI:** 10.1128/jvi.00438-23

**Published:** 2023-04-12

**Authors:** Yukari Itakura, Koshiro Tabata, Takeshi Saito, Kittiya Intaruck, Nijiho Kawaguchi, Mai Kishimoto, Shiho Torii, Shintaro Kobayashi, Naoto Ito, Michiko Harada, Satoshi Inoue, Ken Maeda, Ayato Takada, William W. Hall, Yasuko Orba, Hirofumi Sawa, Michihito Sasaki

**Affiliations:** a Division of Molecular Pathobiology, International Institute for Zoonosis Control, Hokkaido University, Hokkaido, Japan; b Division of Global Epidemiology, International Institute for Zoonosis Control, Hokkaido University, Hokkaido, Japan; c Laboratory of Public Health, Faculty of Veterinary Medicine, Hokkaido University, Hokkaido, Japan; d Laboratory of Zoonotic Diseases, Faculty of Applied Biological Sciences, Gifu University, Gifu, Japan; e Department of Veterinary Science, National Institute of Infectious Diseases, Tokyo, Japan; f National Virus Reference Laboratory, School of Medicine, University College of Dublin, Dublin, Ireland; g International Collaboration Unit, International Institute for Zoonosis Control, Hokkaido University, Hokkaido, Japan; h Global Virus Network, Baltimore, Maryland, USA; i One Health Research Center, Hokkaido University, Hokkaido, Japan; j Institute for Vaccine Research and Development (HU-IVReD), Hokkaido University, Hokkaido, Japan; University of Kentucky College of Medicine

**Keywords:** ESCRT, L-domain, TSG101, matrix protein, rabies virus, rhabdovirus

## Abstract

Viral protein assembly and virion budding are tightly regulated to enable the proper formation of progeny virions. At this late stage in the virus life cycle, some enveloped viruses take advantage of the host endosomal sorting complex required for transport (ESCRT) machinery, which contributes to the physiological functions of membrane modulation and abscission. Bullet-shaped viral particles are unique morphological characteristics of rhabdoviruses; however, the involvement of host factors in rhabdovirus infection and, specifically, the molecular mechanisms underlying virion formation are not fully understood. In the present study, we used a small interfering RNA (siRNA) screening approach and found that the ESCRT-I component TSG101 contributes to the propagation of rabies virus (RABV). We demonstrated that the matrix protein (M) of RABV interacts with TSG101 via the late domain containing the PY and YL motifs, which are conserved in various viral proteins. Loss of the YL motif in the RABV M or the downregulation of host TSG101 expression resulted in the intracellular aggregation of viral proteins and abnormal virus particle formation, indicating a defect in the RABV assembly and budding processes. These results indicate that the interaction of the RABV M and TSG101 is pivotal for not only the efficient budding of progeny RABV from infected cells but also for the bullet-shaped virion morphology.

**IMPORTANCE** Enveloped viruses bud from cells with the host lipid bilayer. Generally, the membrane modulation and abscission are mediated by host ESCRT complexes. Some enveloped viruses utilize their late (L-) domain to interact with ESCRTs, which promotes viral budding. Rhabdoviruses form characteristic bullet-shaped enveloped virions, but the underlying molecular mechanisms involved remain elusive. Here, we showed that TSG101, one of the ESCRT components, supports rabies virus (RABV) budding and proliferation. TSG101 interacted with RABV matrix protein via the L-domain, and the absence of this interaction resulted in intracellular virion accumulation and distortion of the morphology of progeny virions. Our study reveals that virion formation of RABV is highly regulated by TSG101 and the virus matrix protein.

## INTRODUCTION

Rabies virus (RABV) is a well-recognized zoonotic virus that causes a fatal neurological disease in mammals. RABV, belonging to the genus *Lyssavirus* of the family *Rhabdoviridae* in the order *Mononegavirales*, possesses a negative-sense single-stranded RNA genome. This genome encodes five viral proteins, the nucleoprotein (N), phosphoprotein (P), matrix protein (M), glycoprotein (G), and large protein (L) (1). During the RABV virion assembly stage, the viral RNA genome is surrounded by RABV N together with RABV P and L to form a ribonucleoprotein (RNP) with a firmly organized helical structure. The assembly of the RNP continues with an interaction with the RABV M, which provides a “skeleton structure,” and the RNP is finally enveloped by the cellular lipid bilayer containing the RABV G (2). The M-condensing RNP structure is essential for the bullet-shaped viral particles that are a characteristic feature of rhabdoviruses.

In terms of budding and viral particle formation, the importance of the RABV M and G has been highlighted. The RABV G forms membrane microdomains, creates bud sites, and pulls viral particles (3). RABV M makes a major contribution to the pushing out and pinching off virions as well as promoting efficient virus budding and particle formation, and it binds to RNPs to enable their assembly beneath the cell membrane (2, 4–7). Although G-deficient RABV forms noninfectious but bullet-shaped particles, M-deficient RABV rarely forms infectious virus and produces filamentous particles (2, 8). Therefore, RABV M is an essential component in the formation of the bullet-shaped particle structure of RABV.

Some enveloped viruses hijack cellular factors, including the endosomal sorting complex required for transport (ESCRT) machinery, to facilitate the budding process. ESCRTs build complexes by sequentially recruiting ESCRT-0, -I, -II, and -III factors during membrane trafficking, e.g., in the multivesicular body sorting pathway (9–11). The general physiological roles of ESCRTs include the deformation and abscission of lipid membranes, which occur via a process similar to that of virus budding (9, 10). Viral proteins carry consensus amino acid sequences, such as the PY, YL, and PTS/AP motifs, which are called late (L-) domains because they play a role in the recruitment of ESCRT factors at the late stages of the viral life cycle. For example, the human immunodeficiency virus Gag protein, the Marburg virus, and Ebola virus VP40 interact with ESCRT-related proteins, such as Alix and TSG101, via their L-domain to promote budding (12–14). However, little is known about the involvement of ESCRTs in rhabdovirus infection, including RABV infection. Recent proteomic profiling has revealed that ESCRT-related factors, such as chmp4B, HSP40, Alix, TSG101, and chmp2A, are present in purified RABV virions (15). In addition, RABV M possesses L-domains consisting of the PY and YL motifs, and a previous study revealed that mutations in the PY motif of RABV M led to both reduced budding efficiency and pathogenicity of RABV in mice (16). However, the molecular mechanisms underlying the ESCRT-mediated virion assembly and budding in RABV-infected cells have not been clarified.

In the present study, TSG101, a member of the ESCRT-I proteins, was identified as a binding partner of RABV M during the late stage of the virus life cycle. We demonstrate that both the PY and YL motifs of RABV M act as a functional L-domain to enable the interaction with TSG101. RABV propagated in the absence of TSG101 or a recombinant RABV mutant lacking the YL motif failed to bud from the cell membrane and instead formed disrupted virions. Overall, our findings indicate that TSG101 and the L-domain of RABV M are responsible for efficient virus budding and the bullet-shaped morphology of RABV.

## RESULTS

### RNA interference (RNAi) screening identifies TSG101 as an ESCRT factor that supports RABV infection.

**FIG 1:**
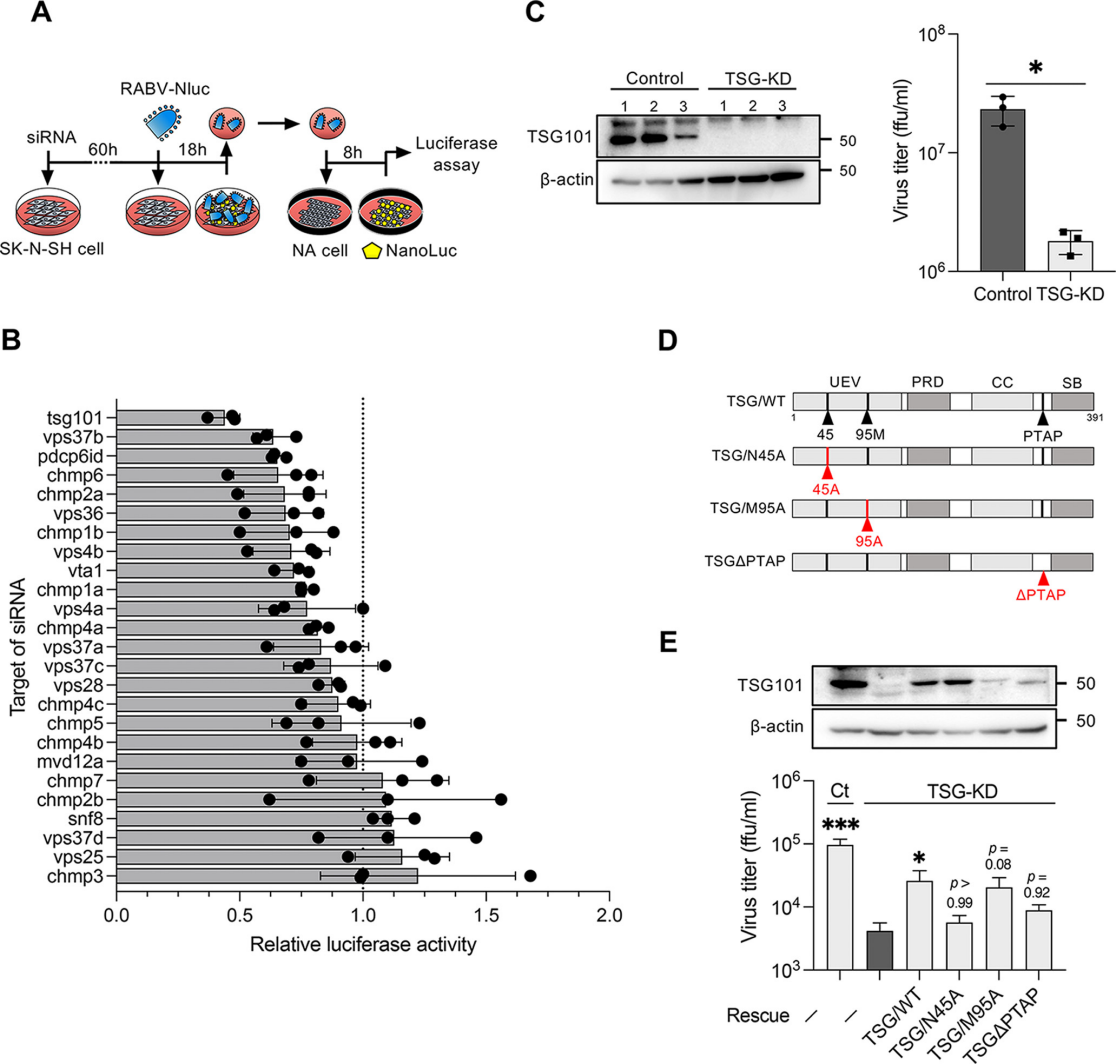
RNAi screening identifies TSG101 as an ESCRT factor supporting RABV infection. (A) Schematic image of the RNAi screening method. siRNA-transfected SK-N-SH cells were infected with RABV-Nluc at an MOI of 10, and culture supernatants collected at 18 hpi were passaged into NA cells. Luciferase activity in the NA cells was measured at 8 hpi. (B) Luciferase activity derived from NanoLuc-encoded reporter RABV in siRNA-treated SK-N-SH cells relative to the luciferase activity in control siRNA-treated cells. Dots indicate the mean of three different siRNAs for each target. Bars indicate the means ± standard deviations of the three siRNAs. (C) Virus titers in the supernatants of TSG-KD SK-N-SH cells at 48 hpi. siRNA-treated cells were infected with RABV at an MOI of 1. The titers were measured using a focus-forming assay. Bars indicate the means ± standard deviations of three replicates from a representative experiment. (D) Schematic images of the TSG101 mutants used in this study. Mutation sites are marked in red. UEV, ubiquitin-conjugating enzyme E2 variant; PRD, proline-rich domain; CC, coiled-coil domain; PTAP, conserved PTAP tetrapeptide motif; SB, steadiness box. (E) Virus titers in TSG-KD and rescue cells. TSG-KD cells were transfected with siRNA-resistant TSG101-encoding plasmids and infected with RABV at an MOI of 1. The virus titers in supernatants at 24 hpi were measured. Bars indicate the means ± standard deviations of three replicates from a representative experiment. For statistical analyses, Welch’s *t* test used in panel C (*, *P < *0.05), and one-way ANOVA and Dunn’s multiple-comparison tests were used in panel E (*, *P < *0.05; ***, *P < *0.001).

To identify the ESCRT factors involved in RABV growth, ESCRT knockdown SK-N-SH cells with a custom small interfering RNA (siRNA) library targeting ESCRT factors were infected with luciferase-expressing RABV, and the progeny virus from the cells was quantified using luciferase assays (Fig. 1A and B). Among 25 ESCRTs and their related factors, the knockdown of *TSG101* was most efficient in terms of decreasing the luciferase signal from progeny RABV in the culture supernatants (Fig. 1B). We confirmed that the knockdown of *TSG101* significantly decreased the progeny virus titer compared with that detected following the control siRNA treatment (Fig. 1C). These results reveal, for the first time, that host TSG101 is a host factor involved in efficient RABV proliferation.

To confirm the TSG101 dependency of RABV infection, we performed a gain-of-function assay by reintroducing TSG101-expressing plasmids into TSG101 knockdown (TSG-KD) cells. In addition to wild-type TSG101, we used three TSG101 mutants, mutants carrying amino acid substitutions at a ubiquitin-binding site (TSG/N45A) or a PT(/S)AP-binding site (TSG/M95A) and a mutant lacking a PTAP motif (TSGΔPTAP), which is involved in recognition by the viral L-domain (Fig. 1D). The exogenous expression of wild-type TSG101 significantly rescued the virus titer in TSG-KD cells. Among the three TSG101 mutants, the expression of the TSG/M95A mutant resulted in the highest rescue efficacy, whereas the expression of the TSG/N45A and TSGΔPTAP mutants only exerted partial effects on viral growth in TSG-KD cells (Fig. 1E). These results indicate that the ubiquitin-binding site and PTAP motif in TSG101 would appear to be required for TSG101-mediated RABV growth.

### Involvement of TSG101 in the assembly and budding processes of RABV virions.

**FIG 2:**
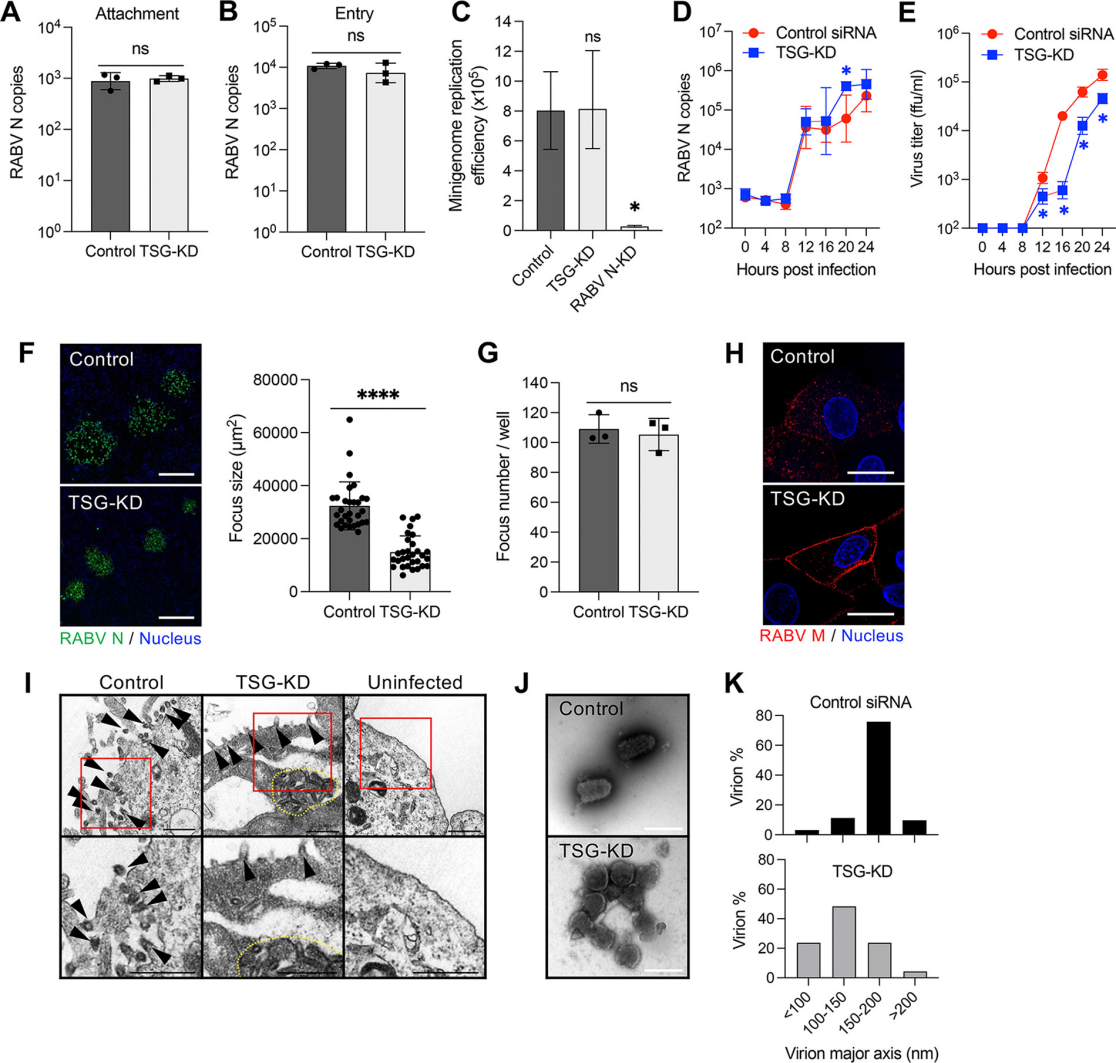
Downregulation of TSG101 expression obstructs the RABV budding process. (A) Virus attachment on the surface of TSG-KD cells. SK-N-SH cells were incubated with RABV at 4°C for 1 h. After the cells were washed, RNA was extracted with attached virions and analyzed using qRT-PCR. (B) Viral entry into TSG-KD cells. SK-N-SH cells were infected with RABV at an MOI of 10. After incubation at 37°C for 30 min, uninternalized virions were removed via trypsin treatment. Internalized virions were measured using qRT-PCR. (C) RABV minigenome replication in TSG-KD cells. 293T cells exogenically expressing the RABV minigenome were transfected with siRNA against TSG101. Minigenome replication was evaluated by measuring the luminescence signal from NanoLuc. (D) Viral RNA levels at the early stage of virus infection. Viral RNA levels in TSG-KD SK-N-SH cells were measured at the indicated time points using qRT-PCR. (E) Virus titers at the early stage of virus infection. Virus titers in the supernatants from TSG-KD SK-N-SH cells were measured at the indicated time points. (F) Focus size of RABV-infected TSG-KD A549 cells. Foci formed by RABV-infected cells were immunostained with anti-RABV N antibody at 72 hpi. Scale bar, 200 μm. The areas of 30 foci selected randomly were measured using ImageJ. (G) Number of foci in TSG-KD A549 cells. (H) Localization of RABV M in TSG-KD SK-N-SH cells. Cells infected with RABV were immunostained with anti-RABV M antibody at 24 hpi and analyzed using confocal microscopy. Scale bar, 20 μm. (I) Electron microscopic images of RABV-infected TSG-KD SK-N-SH cells at 28 hpi. Arrowheads, virions; dotted line, accumulation of virions. Scale bar, 500 nm. (J) Purified RABV virions were negatively stained and analyzed using electron microscopy. Scale bar, 200 nm. (K) Virion diameter and abundance ratio. Purified RABV virions in 50 images captured randomly with an electron microscope were measured using ImageJ. (A to G) Means ± standard deviations of three replicates from a representative experiment. Statistical analyses in panels A, B, F, and G were performed by Welch’s *t* test (*, *P < *0.05; ****, *P < *0.0001); those in panel C were performed by one-way ANOVA and Dunnett’s multiple-comparison test (*, *P < *0.05); and those in panels D and E were done by multiple *t*-tests (*, *P < *0.05). ns, not significant.

Next, we attempted to determine which steps in the RABV life cycle are promoted by TSG101 expression. TSG-KD had no effect on the cellular attachment and entry steps of RABV infection (Fig. 2A and B). A minigenome assay showed that replication and gene expression from a RABV minigenome were not affected by the suppression of TSG101 expression (Fig. 2C). Moreover, intracellular viral RNA levels were similar in control and TSG-KD cells until 24 h postinfection (hpi) (Fig. 2D). Infectious virions were detected from 12 hpi in both cell lines. The virus titer increased markedly at 16 hpi in control cells, whereas a similar increase occurred 4 h later (i.e., 20 hpi) in TSG-KD cells (Fig. 2E). Additionally, the focus size of RABV-infected TSG-KD cells was significantly decreased compared to that of control cells (Fig. 2F), in contrast to the number of RABV-infected foci, which was identical under both conditions (Fig. 2G). The accumulation of RABV M and virions was observed beneath the cell membrane using confocal and electron microscope imaging (Fig. 2H and I). Notably, viral particles released from TSG-KD cells had a rounded form, which is distinct from the typical bullet shape of RABV virions (Fig. 2J). The length of the major axis was 150–200 nm in wild-type RABV virions, but the length shifted to 100–150 nm in progeny virions released from TSG-KD cells (Fig. 2K). These results indicate that TSG101 plays a role in the late stage of RABV infection, specifically in virion formation and release as well as subsequent spread to neighboring cells.

### TSG101 interacts with the L-domain in RABV M.

**FIG 3:**
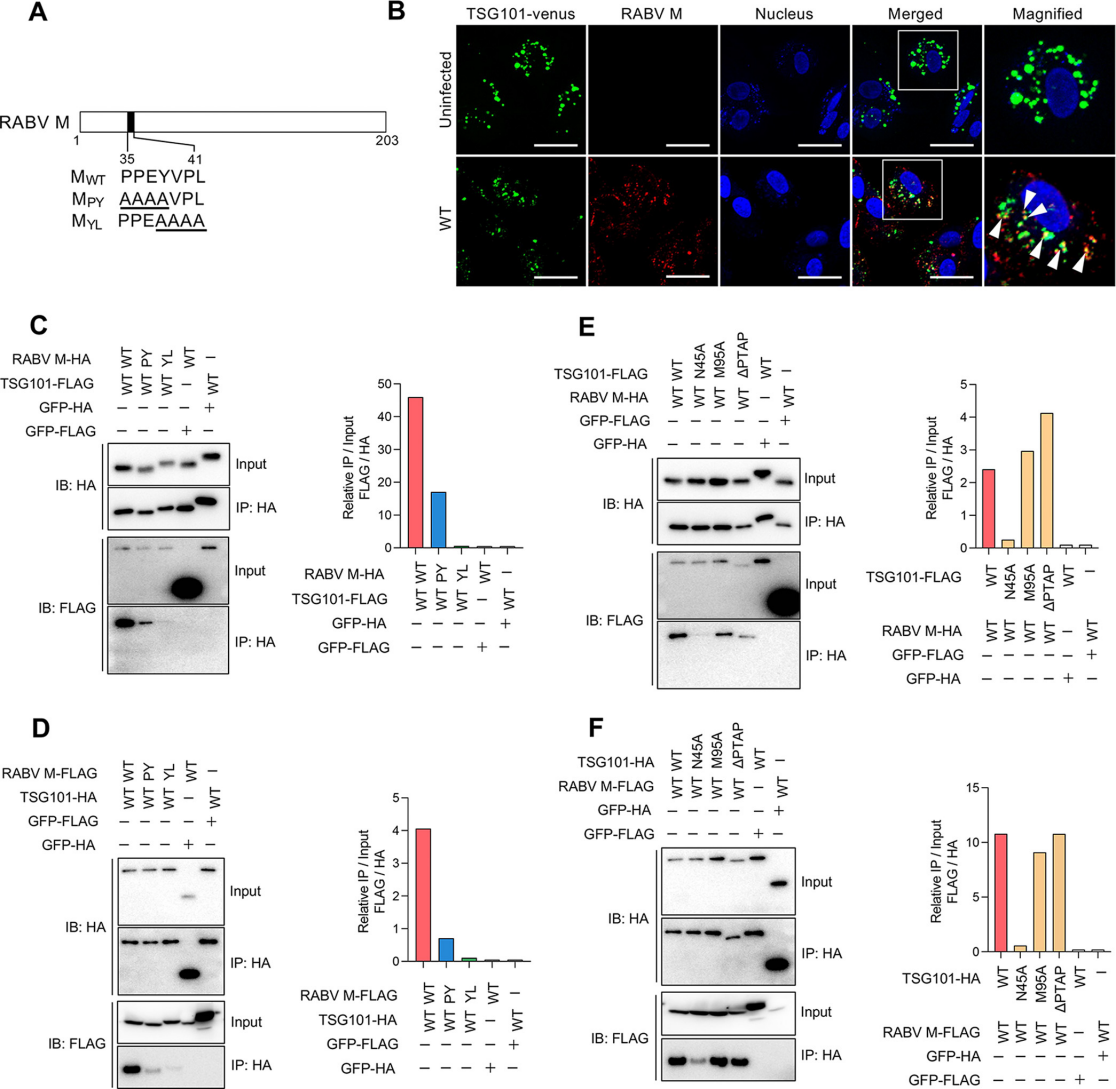
RABV M interacts with TSG101 via the L-domain. (A) Schematic representation of RABV M and the L-domain mutants. (B) Colocalization of RABV M and TSG101. SK-N-SH cells stably expressing TSG101-Venus were infected with RABV, immunostained with anti-RABV M at 24 hpi, and analyzed using confocal microscopy. Arrowheads show colocalization. Scale bar, 50 μm. (C to F) Coimmunoprecipitation of RABV M with TSG101. HA- or FLAG-tagged RABV M and TSG101 were coexpressed in 293T cells and coimmunoprecipitated using anti-HA magnetic beads. Immunoblotting was performed with anti-HA or -FLAG antibody. Bar graphs show the relative precipitation efficacy (IP/input) of FLAG compared with that of HA from a representative experiment following quantification via ImageJ.

TSG101 has been reported to interact with the L-domain in the viral proteins of some envelope viruses (12, 14, 17, 18). RABV possesses two representative L-domains, PY (PPEY) and YL (YVPL) motifs at amino acid positions 35–38 and 38–41 in the RABV M, respectively (Fig. 3A). Therefore, we examined the interaction between RABV M and TSG101. In SK-N-SH cells infected with RABV, the RABV M protein sparsely colocalized with exogenously expressed TSG101-Venus fusion protein (Fig. 3B). Additionally, when RABV M and TSG101 were coexpressed in 293T cells, they were also shown to interact in coimmunoprecipitation (co-IP) studies (Fig. 3C to F). To identify the region responsible for the interaction between RABV M and TSG101, we included RABV M and TSG101 mutants in the co-IP assays. RABV M mutants with a substitution in each L-domain (Fig. 3A) showed lower binding affinity to TSG101 (Fig. 3C and D). In particular, the lack of the YL motif in RABV M led to a marked decrease in the interaction between RABV M and TSG101 (Fig. 3C and D). Additionally, TSG/N45A (the ubiquitin binding-deficient mutant) showed a lower interaction with RABV M (Fig. 3E and F). These results suggest that RABV M interacts with TSG101, and their respective L-domain and ubiquitin-binding site are the likely target sites for the interactions.

Furthermore, the PY and YL motifs in RABV M are conserved throughout the lineages of RABV (see Fig. S1A in the supplemental material). As well as the fixed virulent challenge virus standard (CVS) strain, the fixed attenuated high egg passage-Flury (HEP) strain and street Toyohashi strain exhibited significantly decreased virus titers in TSG-KD cells (Fig. S1B), suggesting a general function of the RABV M-TSG101 interaction in RABV infection.

### Involvement of other ESCRT-related factors and binding mode of TSG101-RABV M.

ESCRT factors sequentially form a complex in their general function of membrane modulations. Besides TSG101, Alix is one of the early-acting proteins in the ESCRT machinery and is known to interact with TSG101 (19). Alix recruits E3 ubiquitin ligase Nedd4 and induces ubiquitination of a binding partner (20). Reportedly, proteins possessing the PY motif are targeted by ubiquitin ligases possessing the WW domain, including Nedd4 and Nedd4-like, while the YL motif is recognized by Alix for its protein interactions (21). Alix, Nedd4, and Nedd4-like were coimmunoprecipitated with RABV M, and depletion of the L-domain in RABV M had little impact on the interactions (Fig. S2A to C). Progeny virus titer was not decreased significantly under the knockdown condition of these factors, suggesting partial contributions in the RABV life cycle (Fig. S2D and E). In contrast, virus growth was suppressed significantly by the knockdown of VPS4 ATPase (Fig. S3A to C), which is the key mediator at the final step of membrane abscission (22). These results indicated that multiple ESCRT-related factors are involved in the complex with RABV M, where some of them are likely to have partial contributions to the function of the machinery.

The binding mode of TSG101-RABV M was further investigated by enzyme-linked immunosorbent assay (ELISA) using recombinant proteins (Fig. S4A). For production of recombinant protein in bacteria, TSG101 was divided into two parts, the N-terminal part (TSG-UEV) and the C-terminal part (TSG-ΔUEV). Recombinant TSG-UEV specifically bound to Alix, but not green fluorescent protein (GFP) control, as reported previously (Fig. S4B and C) (19). Interestingly, recombinant RABV M bound to both TSG-UEV and TSG-ΔUEV, but not GFP control (Fig. S4D and E). Recombinant RABV M also bound to Alix, which was consistent with the result of co-IP (Fig. S2A, S4F). These results suggested that RABV M has the potential to interact directly with TSG101 and Alix.

### Mutation at the L-domain disrupts the TSG101-dependent infection of RABV.

**FIG 4:**
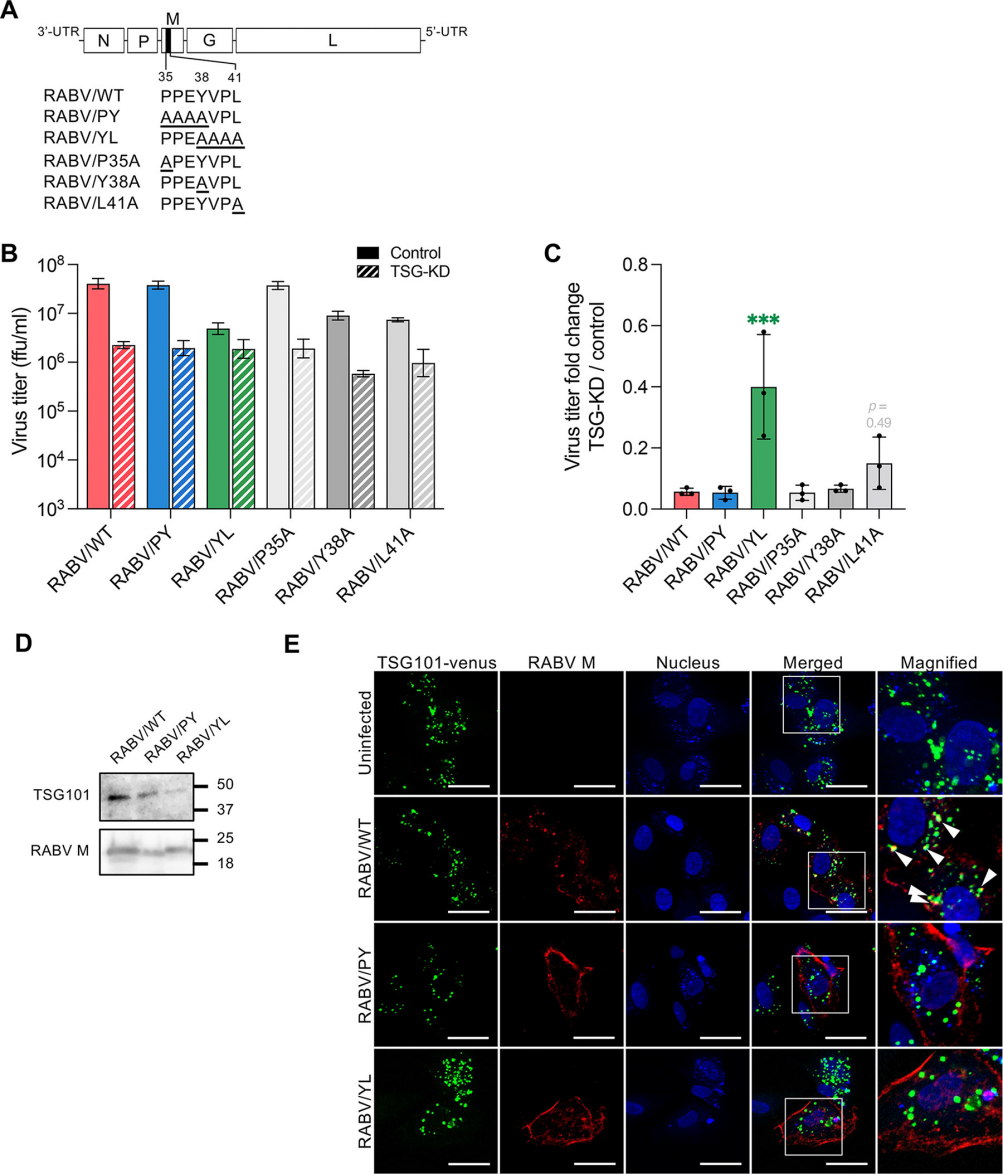
The RABV YL motif is essential for TSG101-mediated viral growth. (A) Schematic representation of recombinant RABV with alanine substitutions in the L-domain in RABV M. (B) Virus titers of RABV L-domain mutants. TSG-KD SK-N-SH cells were infected with RABV mutants at an MOI of 1, and virus titers in the supernatants at 48 hpi were measured. (C) Fold change of virus titers of RABV mutants compared to that of wild-type virus. Means ± standard deviations of three replicates from a representative experiment. Ordinary one-way ANOVA with Dunnett’s multiple-comparison test; ***, *P* < 0.001. (D) Immunoblotting of TSG101 in purified virions. (E) Colocalization of RABV M and TSG101. SK-N-SH cells stably expressing TSG101-Venus were infected with RABV, immunostained with anti-RABV M at 24 hpi, and analyzed using confocal microscopy. Arrowheads show colocalization. Scale bar, 50 μm.

To further determine the importance of the RABV L-domain in the virus life cycle, we generated replication-competent RABV mutant clones (Fig. 4A). Since the PY and YL motifs share the residue Y at position 38 in RABV M, we generated infectious clones with a single amino acid mutation either at P35, Y38, or L41 in RABV M to clarify the importance of each motif. Whole residues in the PY and YL motifs of RABV M were substituted with alanine in RABV/PY and RABV/YL mutants, respectively. Single and multiple amino acid mutations in the YL motif of RABV M reduced the progeny virus titers in SK-N-SH cells (Fig. 4B). We also found that TSG101-KD showed a limited effect on the proliferation of the RABV/YL and RABV/L41A mutants compared to wild-type (WT) virus (Fig. 4C). Given the low sensitivity of TSG-KD, we further characterized RABV/YL mutant in comparison with the RABV/WT and RABV/PY. The amount of TSG101 contained in virions was decreased for RABV/PY and RABV/YL mutants compared to RABV/WT (Fig. 4D). The accumulation of RABV M in the peripheral cytoplasm, which was observed in TSG-KD cells infected with RABV/WT (Fig. 2H), was reproduced in control cells infected with RABV/PY and RABV/YL (Fig. 4E). In contrast to sparse colocalization between RABV M and TSG101-Venus, RABV/PY M and RABV/YL M did not colocalize with TSG101-Venus in the infected cells (Fig. 4E). These results suggest that both the PY and YL motifs in the RABV L-domain could mediate the RABV M-TSG101 interaction during viral infection, and the YL motif is more important in the TSG101-dependent RABV life cycle.

### Mutation at the L-domain perturbates RABV budding and spread *in vitro*.

**FIG 5:**
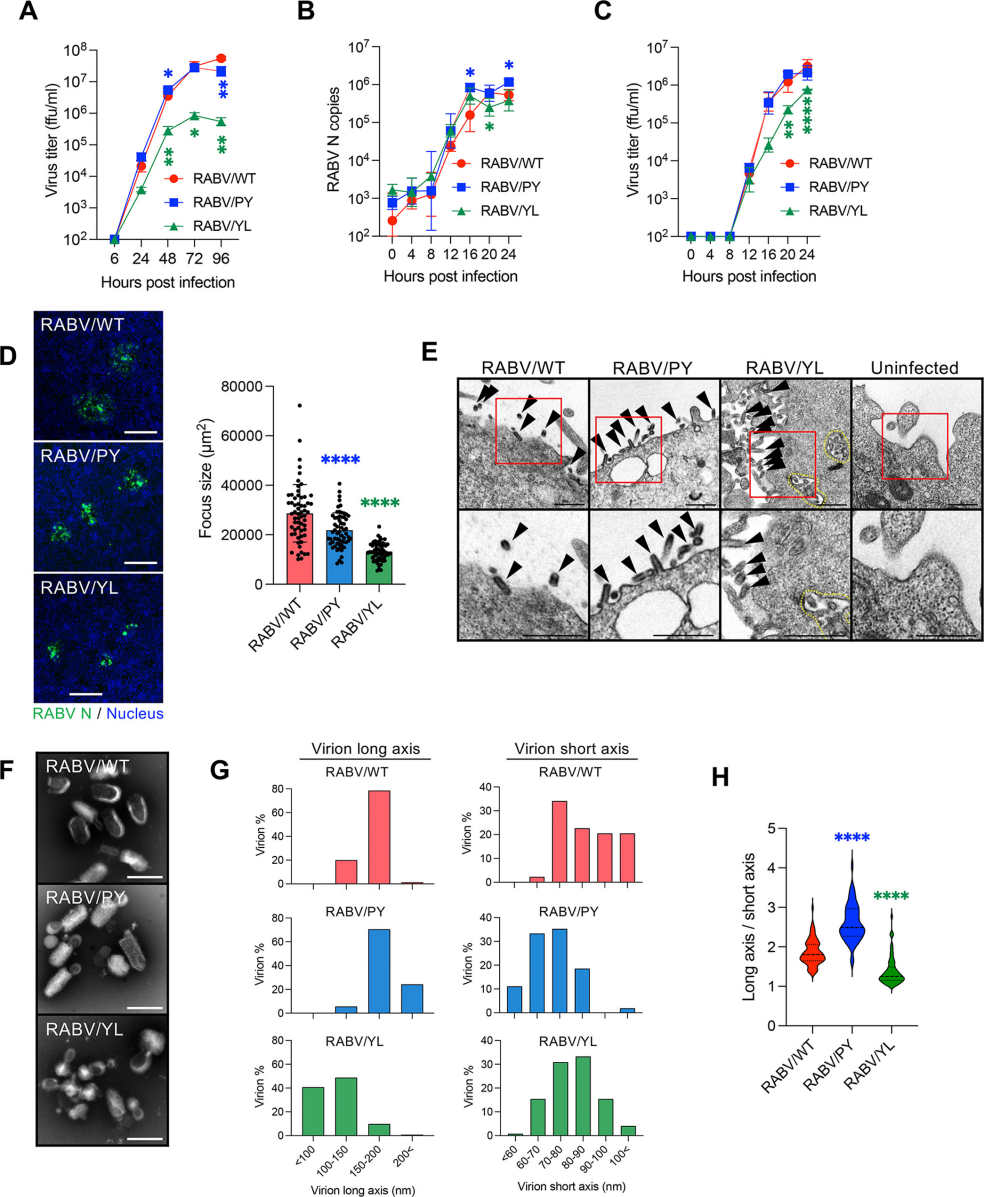
Impaired growth of RABV L-domain mutants. (A) Virus growth curves in SK-N-SH cells during a multicycle infection. Cells were infected with RABV at an MOI of 0.01, and virus titers in the supernatants were measured at the indicated time points. (B) Viral RNA levels at the early stage of virus infection in SK-N-SH cells. Cells were infected with RABV at an MOI of 1, and virus replication in the cells was evaluated at the indicated time points using qRT-PCR. (C) Virus titers at the early stage of virus infection in SK-N-SH cells. Cells were infected with RABV at an MOI of 1, and virus titers in the supernatants were measured at the indicated time points. (D) Focus size of RABV L-domain mutants at 50 hpi. The areas of 60 foci selected randomly in NA cells were measured using ImageJ. Scale bar, 200 μm. (E) Electron microscopy images of SK-N-SH cells at 28 hpi of RABV L-domain mutants. Arrowheads, virions; dotted line, accumulation of virions. Scale bar, 500 nm. (F) Purified virions of RABV L-domain mutants were negatively stained and analyzed using electron microscopy. Scale bar, 200 nm. (G, H) Purified RABV virions in 50 images captured randomly with an electron microscope were measured using ImageJ. (G) Abundance ratio of virion diameter in long axis and short axis. (H) Ratio of long axis to short axis of virions. The middle dotted lines indicate median; top and bottom dotted lines indicate quartile. (A to C) Means ± standard deviations of three replicates from a representative experiment. Statistical analyses in panels A to C were performed by multiple *t* tests (*, *P < *0.05; **, *P < *0.01; ****, *P < *0.0001). Those in panels D and H were performed by one-way ANOVA and Dunnett’s multiple-comparison test (****, *P < *0.0001).

RABV/PY and RABV/YL were further characterized to better understand the role of the L-domain in RABV infection. In SK-N-SH cells, the viral growth of RABV/PY was comparable with that of RABV/WT, whereas the growth efficiency of RABV/YL was lower than that of RABV/PY and RABV/WT (Fig. 5A). RABV/WT and the two mutants showed similar trends in viral RNA replication until 24 hpi (Fig. 5B). Although the infectious progeny viruses of RABV/WT and the mutants were detected in the culture supernatants from 12 hpi, RABV/YL showed a considerably lower virus titer after 16 hpi (Fig. 5C). In terms of focus formation, the focus sizes of RABV/PY and RABV/YL were significantly smaller than that of RABV/WT (Fig. 5D), indicating a low efficiency of virus spread by both mutants. Focus size decreased by all patterns of the single amino acid substitutions, and RABV/L41A formed the smallest focus, similar to RABV/YL mutants (Fig. S5A). These results indicate that substitutions at the L-domain of RABV M critically decrease viral proliferation. Accompanied by the accumulation of the RABV M along the plasma membrane (Fig. 4E), virus particles remained tethered to the cell surface in RABV/PY and RABV/YL infection, as observed under electron microscopy (Fig. 5E). Additionally, the accumulation of virions in the cytoplasm of RABV/YL-infected cells was observed (Fig. 5E). In terms of virion morphology, RABV/PY had slightly thinner and longer virions than those of RABV/WT, whereas RABV/YL formed rounded particles that clearly differed from the representative bulletlike particles of rhabdoviruses (Fig. 5F to H). In addition, RABV/P35A formed thinner and longer virions like RABV/PY, and RABV/L41A made rounded virions like RABV/YL (Fig. S5B and C). P35 and L41 are likely to be the most critical amino acid residues for the function of PY and YL motifs, respectively. Notably, the characteristics of RABV/YL were consistent with those of RABV/WT in TSG-KD cells (Fig. 2D to K). These results suggest that the L-domain, especially the YL motif, in the RABV M plays an essential role in RABV budding and bulletlike virus particle formation.

### Pathogenicity of RABV L-domain mutants *in vivo*.

**FIG 6:**
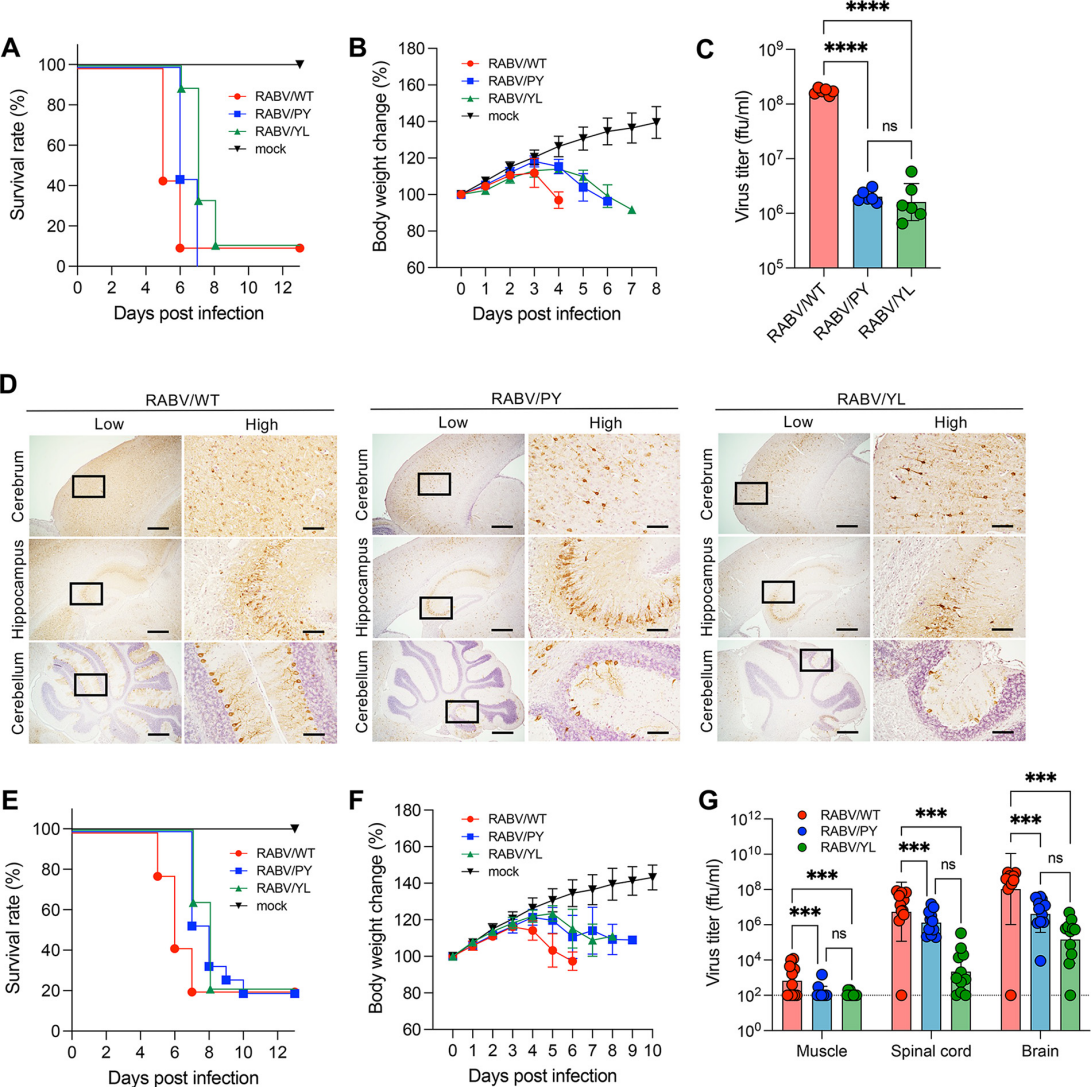
Attenuation of RABV L-domain mutants *in vivo*. Five-week-old ddY mice were inoculated with 10^2^ or 10^5^ FFU RABV intracranially (A to D) or intramuscularly (E to G). Virus-infected mice were monitored for survival (A, E) and body weight changes (B, F). Data in the graphs are means ± standard deviations (mock, *n* = 6 [i.c.], *n* = 6 [i.m.]; RABV-WT, *n* = 8 [i.c.], *n* = 14 [i.m.]; RABV-PY, *n* = 9 [i.c.], *n* = 15 [i.m.]; RABV-YL, *n* = 9 [i.c.], *n* = 14 [i.m.]). (C, G) Virus titers in tissue homogenates at 4 dpi (i.c.) or 5 dpi (i.m.) were determined using a focus-forming assay. Bars show means ± standard deviations (*n* = 6 [i.c.] and *n* = 11 [i.m.] for each group). (D) Immunohistochemistry of the mouse brain. Brain sections at 4 dpi (i.c.) were stained with anti-RABV N. Scale bars, 500 μm (low magnification) and 100 μm (high magnification). Statistical analyses in panels C and G were performed by one-way ANOVA and Tukey’s multiple *t* tests; ***, *P < *0.001; and ****, *P < *0.0001.

To determine the influence of the L-domain on RABV pathogenicity *in vivo*, mice were intracranially (i.c.) or intramuscularly (i.m.) infected with the RABV L-domain mutants. Intracerebral inoculation showed a 1- and 2-day delay in the survival curve and body weight loss for RABV/PY and RABV/YL mutants, respectively (Fig. 6A and B). The virus titers of both mutants in the brain at 4 days postinfection (dpi) were similar, and they were ~80-fold lower than that observed with the parental RABV/WT (Fig. 6C). Viral proteins were distributed extensively across the whole brain of RABV/WT-infected mice, whereas infection was limited in the brains of mice infected with RABV/PY and RABV/YL (Fig. 6D; Fig. S6). Even at 6 dpi, virus growth and distributions of RABV/PY and RABV/YL were limited compared to those of RABV/WT at 4 dpi, whereas the mice were on similar disease progressions (Fig. S7; Fig. 6C). Following intramuscular inoculation, both mutants had a 2-day delay in the survival curve and body weight change compared with the control (Fig. 6E and F). In terms of virus propagation, the virus titer in the brain was 34.8-fold lower for RABV/PY and 509.6-fold lower for RABV/YL than the control virus titer (Fig. 6G). Although the disease progression of mice infected with either mutant was similar, there was an approximate 15 times difference between the virus titers of the two mutants (Fig. 6G). These results suggest that the substitution of the L-domain results in the attenuation of RABV, indicating the importance of both the PY and YL motifs in RABV replication, not only *in vitro* but also *in vivo*.

## DISCUSSION

Virus budding is a sophisticated process required for infectious viral particle production and release. In rhabdovirus infection, the M protein is essential for budding and the formation of typical bullet-shaped particles, given that M-deficient viruses form long filamentous particles that are not infective (2). RABV M also has multiple functions at the late stage of RABV infection, when it is involved in the condensation and assembly of RNPs, as well as the pushing out and pinching off of viral particles (2–7, 23). However, little is known about the host factors involved in the budding and particle formation processes of rhabdoviruses. Our RNAi screen identified TSG101 as an ESCRT factor that supports RABV infection. Moreover, our experiments demonstrated that TSG101 facilitates RABV budding and virion formation.

The ESCRTs play roles in membrane scission, which is a crucial process in cytokinesis (9, 11) and the formation of endosomal luminal vesicles and exosomes (10). ESCRT-mediated membrane invagination and vesicle formation is a process analogous to the budding step during the release of virions from cells. Some enveloped viruses, such as retroviruses, filoviruses, paramyxoviruses, and flaviviruses, are reported to use the ESCRT in viral particle budding (12, 14, 17, 18). In the present study, RABV growth was significantly impaired by TSG-KD (Fig. 1C); however, the knockdown of TSG101 had no effect on viral entry, genome replication, and protein synthesis (Fig. 2A to D), although it caused intracellular accumulation of viral particles and the distortion of virion morphology (Fig. 2H to K). Since virions tethered to the cell surface form bullet-shaped particles (Fig. 2I), the later process of pinch-off or stability of virions might be disturbed by TSG-KD. These results provide evidence that TSG101 contributes to the late stage of RABV infection, specifically virion assembly and budding. Nevertheless, given the multiple roles of TSG101 in cellular homeostasis, we cannot exclude the possibility that TSG101 is involved more extensively in the RABV life cycle.

L-domains are consensus amino acid sequences observed in the structural proteins of various viruses and are known to be involved in the recruitment of ESCRTs (24). RABV possesses an L-domain composed of overlapping PY and YL motifs (PPEYVPL) at amino acid positions 38 to 41 in RABV M. Amino acid substitutions of the PY motif in RABV M are reported to decrease the growth and pathogenicity of RABV; however, the molecular mechanisms underlying this attenuation remain to be elucidated (16). In the current study, we have demonstrated that TSG101 interacts with RABV M via the L-domain, which is required for the colocalization of these proteins (Fig. 3B and Fig. 4E). Recombinant RABVs lacking the L-domain in RABV M show low viral proliferation and pathogenicity both *in vitro* and *in vivo* (Fig. 5A; Fig. 6C and G). Notably, the RABV/YL mutant showed an impaired budding ability leading to an abnormal particle morphology (Fig. 5F). The residue L41 especially seems responsible for the features driven by the YL motif. Meanwhile, the lack of a PY motif increased the viral particle length (Fig. 5F to H), suggesting that PY motif may contribute to budding and virion formation of RABV through a mechanism different from that of the YL motif. Collectively, our results revealed an important function of the L-domain in the TSG-dependent RABV life cycle. In contrast, vesicular stomatitis virus (VSV), a prototype rhabdovirus, does not possess the YL motif, but it has the PY motif and another L-domain, the PSAP motif in VSV M. Use of ESCRTs in VSV budding is controversial since both its dependency and independency on ESCRTs have been reported (25–27).

In the present study, we found that RABV M directly interacts with both TSG-UEV and TSGΔUEV, suggesting possible multiple binding sites (Fig. S4B). In addition to constructing complexes with other ESCRTs, the key functions of TSG101 in endocytic trafficking are mediated by binding to one of the L-domains, i.e., to P(T/S)AP motif-containing proteins via methionine at position 95 (95M) or to ubiquitin via asparagine at position 45 (45N) (11, 28–30). None of the RABV proteins possess the P(T/S)AP motif, and our rescue experiments (Fig. 1E) and immunoprecipitation assays (Fig. 3E and F) suggest that 95M in TSG101 is not required for RABV replication. However, the introduction of an amino acid mutation on 45N (TSG/N45A) resulted in a poor RABV growth rescue rate (Fig. 1E) and a weakened RABV M-binding capacity (Fig. 3E and F). Considering the role played by ubiquitin in triggering cell membrane invagination for endocytosis (31), the involvement of ubiquitin in virus budding appears plausible (14, 20, 32–34). During rhabdovirus infection, M is also ubiquitinated by the host E3 ubiquitin ligase (32, 33). Even though we assessed the direct interaction between recombinant RABV M and TSG101, we cannot exclude the possibility of contamination of ubiquitinated RABV M in the recombinant protein used for the ELISA. Thus, based on the present data and previous studies, we speculate that the ubiquitination of RABV M, the Alix-RABV M interaction, or involvement of Nedd4 might facilitate the RABV M-TSG101 interaction through the protein complex with its capacity to interact with various elements.

In summary, this study has revealed that TSG101 supports the budding and formation of RABV viral particles through its interaction with RABV M via the L-domain. Considering the involvement of host factors in RABV infection, the discovery of a host factor that plays a critical role in the egress of RABV provides new insights into the mechanisms of RABV budding and virion formation, especially as it highlights the importance of the L-domain in RABV M. In future studies, focusing on the involvement of other ESCRT factors and ubiquitin will improve our understanding of the mechanisms underlying RABV budding and the production of bullet-shaped virion formation and may help integrate previously disconnected studies.

## MATERIALS AND METHODS

### Ethics statement.

Animal experiments were approved by the Institutional Animal Care and Use Committee of Hokkaido University (approval number 19-0014) and performed according to the committee’s guidelines.

### Cells.

Human neuroblastoma (SK-N-SH) cells, mouse neuroblastoma (NA) cells, and baby hamster kidney cells stably expressing T7 RNA polymerase (BHK/T7-9) cells (35) were maintained in Eagle’s minimum essential medium (MEM) supplemented with 10% fetal bovine serum (FBS). SK-N-SH cells were cultured in type I collagen-coated plates, whereas 293T cells were cultured in Dulbecco’s modified Eagle’s medium supplemented with 10% FBS. All cells were incubated at 37°C in the presence of 5% CO_2_.

SK-N-SH cells stably expressing TSG101-Venus were established using lentivirus transduction. cDNA fragments encoding TSG101 fused at its N terminus with Venus following double-GGGGS linker sequences were subcloned into the pLVSIN-CMV Pur vector. The resulting plasmid was transfected into 293T cells using a lentiviral high-titer packaging mix (TaKaRa) to obtain the lentiviral vector for the TSG101-Venus transduction. The SK-N-SH cells were then infected with the lentivirus vector and selected using puromycin.

### Viruses.

Recombinant RABV (rRABV) clones of the CVS strain, HEP strain, and a reporter CVS encoding NanoLuc (CVS-Nluc [N-P]) were prepared as described previously (36, 37). Recombinant RABV/PY, RABV/YL, RABV/P35A, RABV/Y38A, and RABV/L41A mutants were obtained from the cDNA clones of the CVS strain by substituting the target amino acid residues for alanine, which was achieved via PCR-based mutagenesis according to procedures described previously (35–37). The street RABV Toyohashi strain was prepared as described previously (38). All viruses were propagated in NA cells.

### Quantitative real-time RT-PCR.

Viral RNA copy numbers were quantified using a Thunderbird Probe one-step qRT-PCR kit (Toyobo) and TaqMan probe/primer sets specifically targeting RABV CVS N (F, 5′-TCG AAT GCT GTC GGT CAT GT-3′; R, 5′-CCG AAG AAT TCC TCT CCC AAA TA-3′; probe, 5′-FAM-CAA TCT CAT TCA CTT TGT TG-MGB-3′). The mRNA abundance of VPS4A and VPS4B were quantified using the one-step TB green PrimeScript PLUS RT-PCR kit (TaKaRa Bio) and the primer sets human VPS4A (F, 5′-TTC TCA GCC TCC TGT GAC CAC T-3′; R, 5′-ACG TCG TTC CAC CGT ATG TTG G-3′) and VPS4B (F, 5′-GGT TCT GGA TTC TGC CAT TAG GC-3′; R, 5′-CCG AAA GTC TGC TTC CGT GAG A-3′). The expression levels of housekeeping genes were quantified using predeveloped TaqMan Assay reagent human ACTB (Applied Biosystems).

### RNAi screen.

A custom siRNA library targeting 25 ESCRT-related factors and consisting of three different siRNAs per target gene was used to rule out the off-target effect (Silencer Select predesigned siRNA; Ambion). SK-N-SH cells were reverse transfected with 20 nM siRNA using Lipofectamine RNAiMAX (Invitrogen) in collagen-coated 96-well plates and then cultured for 60 h. Subsequently, the cells were infected with CVS-Nluc (N-P) at a multiplicity of infection (MOI) of 10. A high MOI was employed to minimize the robustness of the assay through optimization. At 18 hpi, the supernatants were transferred into NA cells seeded on 96-well clear-bottom black plates. After 8 h of incubation at 37°C, luminescence signals were measured using a Nano-Glo luciferase assay (Promega) following the manufacturer’s protocol.

### Gene knockdown and virus infection.

SK-N-SH cells were reverse transfected with 20 nM siRNA using Lipofectamine RNAiMAX (Invitrogen) in collagen-coated 48-well plates and then cultured for 60 h. Knockdown efficacy was checked by immunoblotting (anti-Alix antibody [catalog no. 92880; Cell Signaling], anti-Nedd4 antibody [catalog no. 2740; Cell Signaling], anti-Nedd4-like antibody [catalog no. 4013; Cell Signaling], and anti-TSG101 antibody [catalog no. 28283-1-AP; Proteintech]) or qRT-PCR. siRNA-treated cells were infected with virus at an MOI of 1, and the supernatants were collected at 48 to 60 hpi.

### Virus titration via a focus-forming assay.

NA cells were infected with serially diluted specimens in a 48-well plate for 1 h and then overlaid with MEM supplemented with 5% FBS, 0.5% methylcellulose, and GlutaMAX (Gibco). After 3 days of incubation, the cells were fixed and stained with fluorescein isothiocyanate (FITC)-labeled anti-RABV N antibody (Fujirebio) as well as 10 μg/mL of Hoechst 33342. Foci were then counted to determine the virus titer in focus-forming units (FFU).

### Rescue experiment.

SK-N-SH cells were first transfected with 20 nM siRNA against TSG101 using Lipofectamine RNAiMAX (Invitrogen). At 48 h posttransfection of siRNA, the cells were transfected with siRNA-resistant TSG101 constructs using Lipofectamine 2000 (Invitrogen). After 24 h of incubation, the cells were infected with CVS at an MOI of 1, and the supernatant was collected for virus titration at 24 hpi.

### Attachment and entry assays.

The attachment and entry assays were conducted following procedures described previously (39). Briefly, for the attachment assay, SK-N-SH cells were infected with RABV at MOI of 10 at 4°C for 1 h and washed with phosphate-buffered saline (PBS) followed by RNA extraction. For the entry assay, cells were infected with RABV at an MOI of 10 at 37°C for 30 min and washed with PBS followed by trypsinization and RNA extraction. The cell-attached virions and internalized virions were analyzed by measuring viral genome copies using qRT-PCR.

### Minigenome assay.

A CVS strain-based minigenome assay was performed according to a previously reported method with some modification (40) and with gene knockdown. Briefly, plasmids encoding CVS N, P, and L and the RABV minigenome were reverse cotransfected into 293T cells in 96-well plates. After 8 h, the cells were transfected with 20 nM siRNA using Lipofectamine RNAiMAX. Following 70 h of incubation, minigenome replication was evaluated by measuring the luminescence of NanoLuc from the minigenome using Nano-Glo (Promega).

### Immunofluorescence assays.

RABV-infected cells were fixed with 10% phosphate-buffered formalin and washed with PBS. Blocking was conducted with 1% bovine serum albumin in PBS for 1 h at room temperature. The cells were then stained with anti-RABV M antibody (catalog no. A54616; Epigentek; 1:150 dilution) in 0.3% BlockAce in 0.005% Tween 20 in PBS (PBST) overnight at 4°C. After two washes with PBST, Alexa Fluor 594-conjugated anti-rabbit IgG antibody (catalog no. A32740; Invitrogen; 1:1,000 dilution) was added to the cells as the secondary antibody. The stained cells were observed using an LSM780 confocal microscope and ZEN software (Zeiss).

### Electron microscopy analysis.

For ultrathin-section electron microscopy, SK-N-SH cells in a collagen-coated dish (10 cm) were infected with rRABV at an MOI of 5 to 10 and cultured for 28 h. The cells were then washed with phosphate buffer (PB), scraped off the plate, and pelleted via centrifugation. The cell pellets were washed, fixed for 30 min with 2.5% glutaraldehyde in PB on ice, and fixed again with freshly prepared fixative at 4°C overnight. The fixed cell pellets were washed with PB and postfixed with 1% osmium tetroxide in PB for 1 h on ice. Subsequent procedures were conducted as described previously (41).

For negative-stain electron microscopy, rRABV purified via ultracentrifugation with a 20% sucrose cushion (134,000 × *g*, 4°C, 2 h) was fixed with 2.5% glutaraldehyde. The fixed virus was placed on an elastic carbon film, ELS-C10 (catalog no. 10-1012; Oken), and stained with 2% phosphotungstic acid solution (pH 5.8). The samples were then examined using an H-7650 electron microscope (Hitachi).

### Immunoprecipitation assay.

First, 293T cells were cotransfected with plasmids encoding FLAG- or hemagglutinin (HA)-tagged TSG101 and RABV M using polyethylenimine. Cell lysates were harvested at 72 h posttransfection and clarified via centrifugation. Immunoprecipitation was then conducted using anti-HA magnetic beads (Pierce) according to the manufacturer’s protocol. The precipitates were extracted into 1× SDS sample buffer containing 2-mercaptethanol and subjected to a standard immunoblotting assay using horseradish peroxidase (HRP)-conjugated anti-HA antibody (catalog no. H6533; Sigma; 1:3,000 dilution) and anti-FLAG antibody (catalog no. A8592; Sigma; 1:3,000 dilution).

### Expression and purification of recombinant proteins.

The cDNA fragments of human Alix, GFP, and human TSG101 were cloned into pCMV vector to obtain pCMV-Alix-His, pCMV-GFP-His, pCMV-TSG101-His, and pCMV-FLAG-TSG101, respectively. PCR-based deletion was performed to obtain pCMV-TSG-UEV-His and pCMV-FLAG-TSG-ΔUEV. The constructed pCMV vectors were transfected into 293T cells with polyethylenimine. Cell lysates were collected at 72 h posttransfection and clarified via centrifugation. The His-tagged proteins were purified using Ni Sepharose Excel (Cytiva) following the manufacturer’s protocol. The FLAG-tagged protein was purified with anti-DYKDDDDK-tag antibody magnetic beads (FujiFilm) according to the manufacturer’s protocol by using 500 μg/μL of FLAG peptide. The expression vector pET15b-RABV M-His was constructed by cloning RABV M into pET15b vector and used to transform BL21 competent cells. The protein expression was induced with 0.1 mM isopropyl-β-d-thiogalactopyranoside (IPTG) at 37°C for 5 h. Cell pellets were lysed by sonication in lysis buffer. Solubilized protein was purified by affinity chromatography using Ni Sepharose Excel resin (Cytiva).

### ELISA.

ELISA plates were coated with the recombinant protein (5 μg/mL) in coating buffer (carbonate-bicarbonate buffer, pH 9.6; Medicago) overnight at 4°C and blocked with 5% skim milk in PBST for 30 min at 37°C. After rinsing with PBS, the analyte of recombinant protein was diluted in PBST (10, 100, or 1,000 nM) for 1 h at 37°C followed by three washes with PBST. Then, plates were treated with the primary antibodies (anti-TSG101 [catalog no. 28283-1-AP; Proteintech], anti-Alix [catalog no. 92880; Cell Signaling], and anti-GFP [catalog no. 600-101-215; Rockland]) for 1h at 37°C and washed 3 times with PBST, followed by the 1-h, 37°C incubation with the HRP-conjugated secondary antibody (HRP-anti-rabbit IgG [catalog no. 7074; Cell Signaling] and HRP-anti-goat IgG [catalog no. sc-2020; Santa Cruz]) and washed 3 times with PBST. The 1-Step Ultra TMB-ELISA substrate (Thermo) was added, and the reaction was stopped by 2 M H_2_SO_4_. The optical density at 450 nm (OD_450_) was measured by an iMark microplate reader (Bio-Rad).

### Animal experiments.

Five-week-old male ddY mice were used in the animal experiments. Mice were inoculated intracranially or intramuscularly with 10^2^ or 10^5^ FFU of rRABV under anesthesia using isoflurane. All mice in the survival groups were observed daily for disease symptoms and body weight changes. The humane endpoint was defined as a 20% decrease in body weight or an inability to reach food or water due to the onset of disease. Mice were euthanized by decapitation under anesthesia, and their tissues were harvested at specific time points for further analysis.

### Immunohistochemistry.

Brain sections were prepared as described previously (36). After antigen retrieval in citrate buffer for 5 min using a pressure cooker, the sections were treated with 0.3% H_2_O_2_ in methanol for 15 min to inactivate endogenous peroxidase. The sections were then treated with 10% goat serum (Nichirei Biosciences) for 1 h at room temperature and incubated at 4°C overnight with the primary antibody against RABV N (code PA321352LA01RAI; Cusabio; 1:2,500 dilution). After three washes with 0.01% PBST, secondary staining was performed with EnVision system, HRP-labeled polymer anti-rabbit (catalog no. K4220; Dako) for 1 h at room temperature. The slides were washed three times with PBST, and a 3,3′-diaminobenzidine (DAB) substrate kit (catalog no. 425011; Nichirei) was used to visualize the immunostaining.

### Statistical analysis.

All statistical analyses were performed using GraphPad Prism 9.2.0. For comparisons of two groups, Welch’s *t* test was used. For comparisons of two groups at multiple time points, a multiple *t* test was performed using the Benjamini, Krieger, and Yekutieli method. For comparisons among more than two groups, one-way or two-way analysis of variance (ANOVA) with Tukey’s, Holm-Sidak’s, Dunnett’s, or Sidak’s multiple-comparison test was used. Data are presented as means ± standard deviations in graphs. *, *P < *0.05; **, *P < *0.01; ***, *P < *0.001; ****, *P < *0.0001.

### Data availability.

This study includes no data deposited in external repositories.
