# Supplementary material for: Morphogenesis of Bullet-Shaped Rabies Virus Particles Regulated by TSG101
Source: J Virol. 2023 Apr 12;97(5):e00438-23. doi: 10.1128/jvi.00438-23 (PMC10231130; doi:10.1128/jvi.00438-23)
Supplement: Supplemental file 1 — Fig. S1 to S7. Download jvi.00438-23-s0001.pdf, PDF file, 7.2 MB [file jvi.00438-23-s0001.pdf]

Supplemental Information

A

	Lineage	Strain name	27-49th amino acid sequence in RABV M	Accession No.
Fix	Cosmopolitan	CVS	YDDD LWLP **PPEYVPL** KELT SKKN	LC325820
	Cosmopolitan	HEP	DGDD LWLP **PPEYVPL** KELT SKKN	AB085828
	Cosmopolitan	ERA	DDDD LWLP **PPEYVPL** KELT SKKN	AB781935
Street	Cosmopolitan	1088	DDDD LWLP **PPEYVPL** KELT SRKN	AB645847
	Arctic-related	Komatsugawa	DDDD LWLP **PPEYVPL** KELT GKKN	LC553558
	Asia	8743THA	DDDD LWLP **PPEYVPL** KELT GKKN	EU293121
	Asia	Toyohashi	DDDD LWLP **PPEYVPL** KELT SKKN	LC619707.1
	Indian Subcontinent	RY2417	DDDD LWLP **PPEYIPL** KELA GKKN	KF154999
	Africa2	DRV-NG11	DDDD LWLP **PPEYVPL** KELT GKKN	KC196743.1
	RAC-SK	PRV ON-99-2	DDDD LWLP **PPEYVPL** KELT GKKN	EU311738
	Bat	SHBRV-18	DDDD LWLP **PPEYIPL** KEIT GKKN	AY705373

B

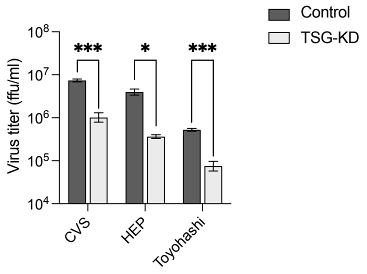

Fig. S1. Conservation and functional importance of the L-domain in various fixed and street strains of RABV.

(A) Table listing the 27th to 49th amino acid sequences in the RABV M. Bold: L-domain; red: strains analyzed in (B). (B) Virus titers of the CVS strain, HEP fixed strain, and Toyohashi street strain in TSG-KD cells. SK-N-SH cells were infected with RABV at a MOI of 1. Virus titers in the culture supernatants at 48 hpi were determined using a focus forming assay. Bars indicate the means ± standard deviations of three replicates from a representative experiment. Statistical analyses: multiple *t*-tests with Welch's correction: *P < 0.05, ***P < 0.001.

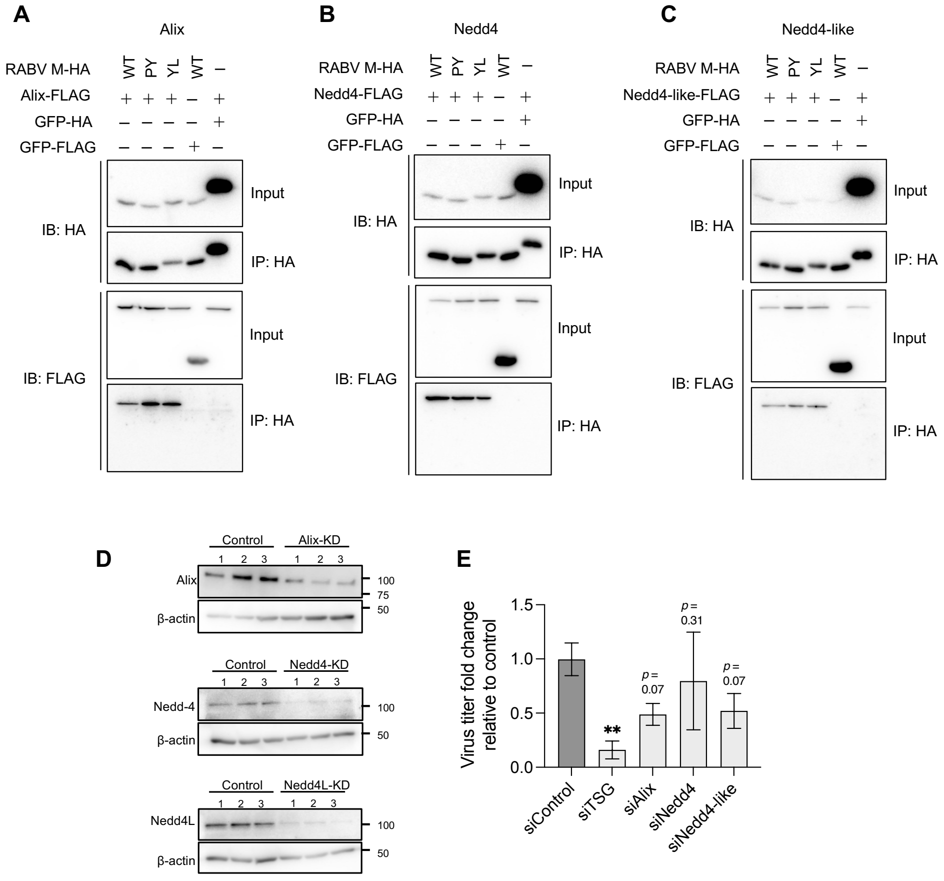

Fig. S2. Interaction of RABV M and ESCRT-related factors.

(A–C) Coimmunoprecipitation of RABV-M with (A) Alix, (B) Nedd4, and (C) Nedd4-like. HA-tagged RABV M and FLAG-tagged partners were coexpressed in 293T cells and coimmunoprecipitated using anti-HA magnetic beads. Immunoblotting was performed with anti-HA or -FLAG antibody. (D) Immunoblotting of Alix, Nedd4, and Nedd4-like in siRNA-treated SK-N-SH cells. (E) Fold change of virus titers in siRNA treated cells compared to control virus titer. SK-N-SH cells were infected with RABV at a MOI of 1. Virus titers in the culture supernatants at 48 hpi were determined using a focus forming assay. Bars indicate the means ± standard deviations of three replicates from a representative experiment. Statistical analyses: Ordinary one-way ANOVA with Holm-Sidak's multiple comparisons test: $^{**}P < 0.01$.

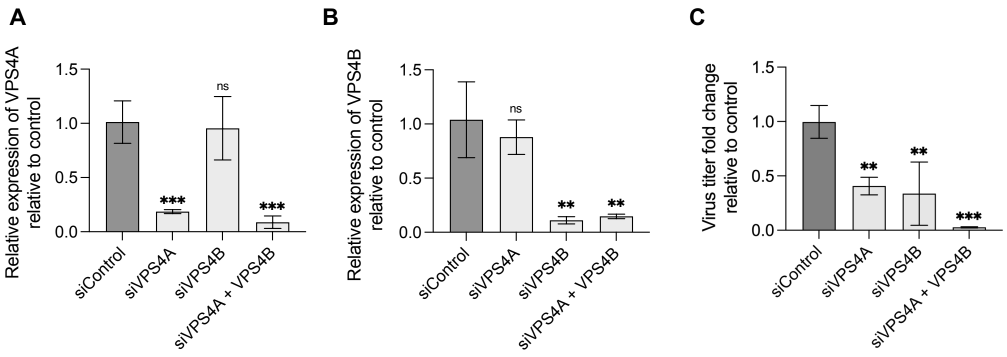

Fig. S3. Knockdown of VPS4 proteins suppressed virus propagation.

(A, B) Suppressed expression of VPS4 by gene knockdown. VPS4A and VPS4B mRNA levels in siRNA-treated SK-N-SH cells were normalized to the β-actin gene and presented as fold changes relative to the control siRNA. (C) Fold change of virus titers in siRNA-treated cells compared to that in control siRNA. SK-N-SH cells were infected with RABV at a MOI of 1. Virus titers in the culture supernatants at 48 hpi were determined using a focus forming assay. Bars indicate the means ± standard deviations of three replicates from a representative experiment. Statistical analyses: Ordinary one-way ANOVA with Holm-Sidak's multiple comparisons test: $^{**}P < 0.01$, $^{***}P < 0.001$.

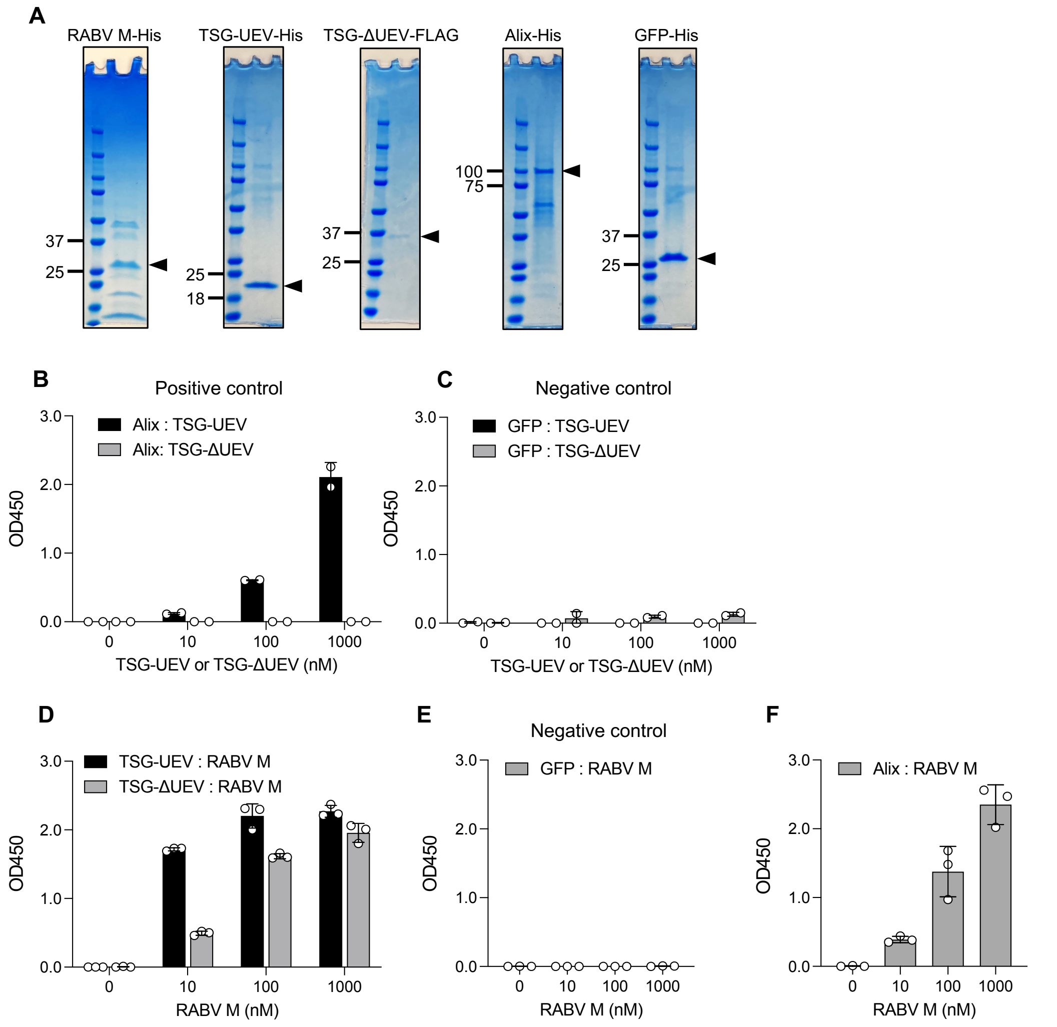

Fig. S4. Direct interaction of RABV M and TSG101 (related to Fig.3).

(A) SDS-PAGE gels showing the molecular weight of purified recombinant proteins. (B–F) ELISA using the purified recombinant proteins. The data are indicated as OD450 adjusted to set the background to zero. Bars indicate the means ± standard deviations of two or three replicates from a representative experiment.

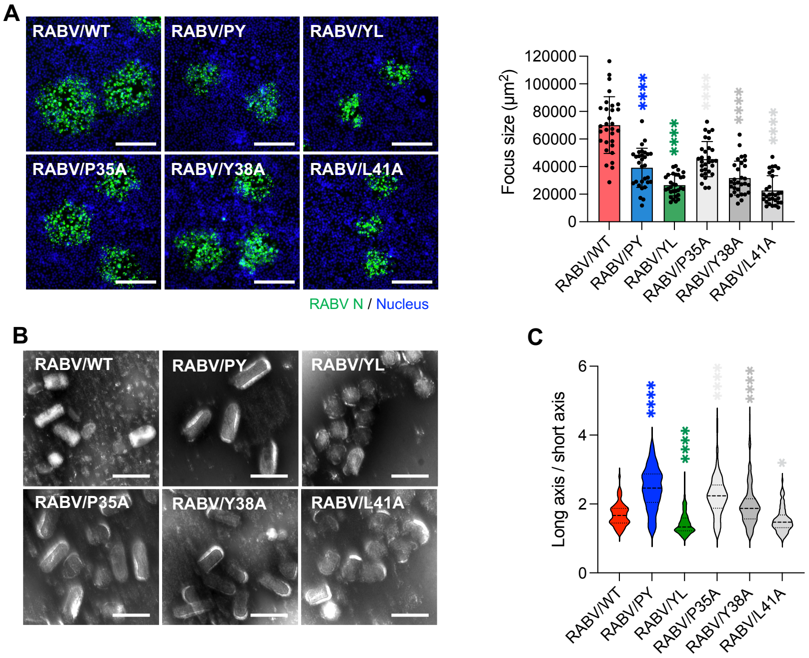

Fig. S5. Characterization of RABV mutants with a single-amino acid substitution at L-domain (related to Fig.5).

(A) Focus size of RABV mutants at 72 hpi. The area of 30 foci selected randomly in NA cells were measured using ImageJ. Scale bar: 200 μm. Means ± standard deviations of three replicates from a representative experiment. (B) Purified virions of RABV mutants negatively stained and analyzed by electron microscopy. Scale bar: 200 nm. (C) Ratio of long axis to short axis of virions. Purified virions in 30 electron micrographs captured randomly were measured using ImageJ. The middle dot lines indicate median, top and bottom dot lines indicate quartile. Statistical analysis: (A, C) Ordinary one-way ANOVA with Dunnett's multiple comparisons test: *P < 0.05, ***P < 0.001, ****P < 0.0001.

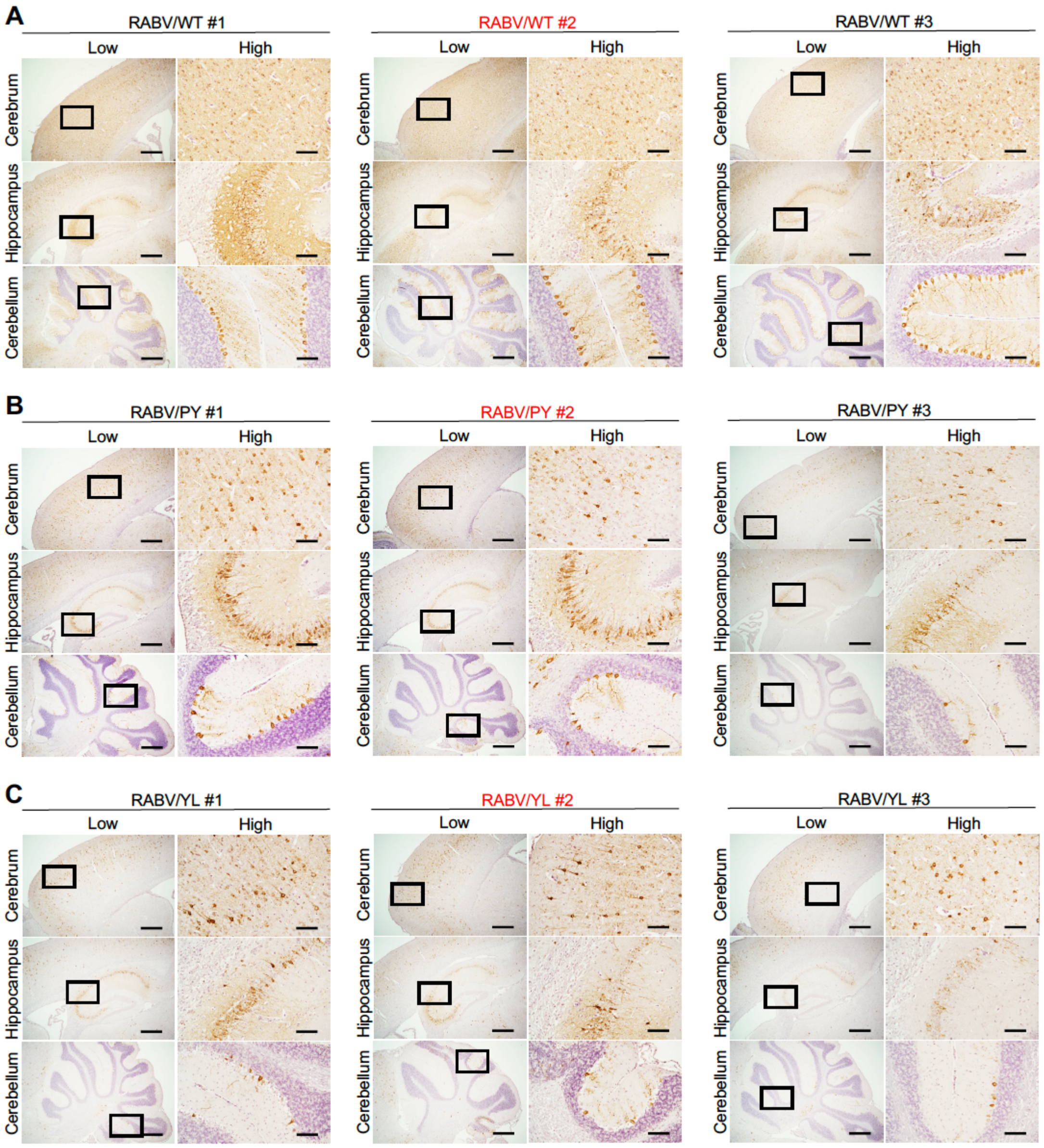

Fig. S6. Distribution of RABV in mouse brains on 4 dpi (related to Fig.6).

Immunohistochemistry of the mouse brain. Five-week-old ddY mice were inoculated with 10^2 ffu RABV intracranially. Brain sections at 6 dpi of (A) RABV/WT, (C) RABV/PY, or (D) RABV/YL were stained with anti-RABV N. Scale bars: 500 μm (low magnification) and 100 μm (high magnification). Red-highlighted data are shown in the main figure as representative images.

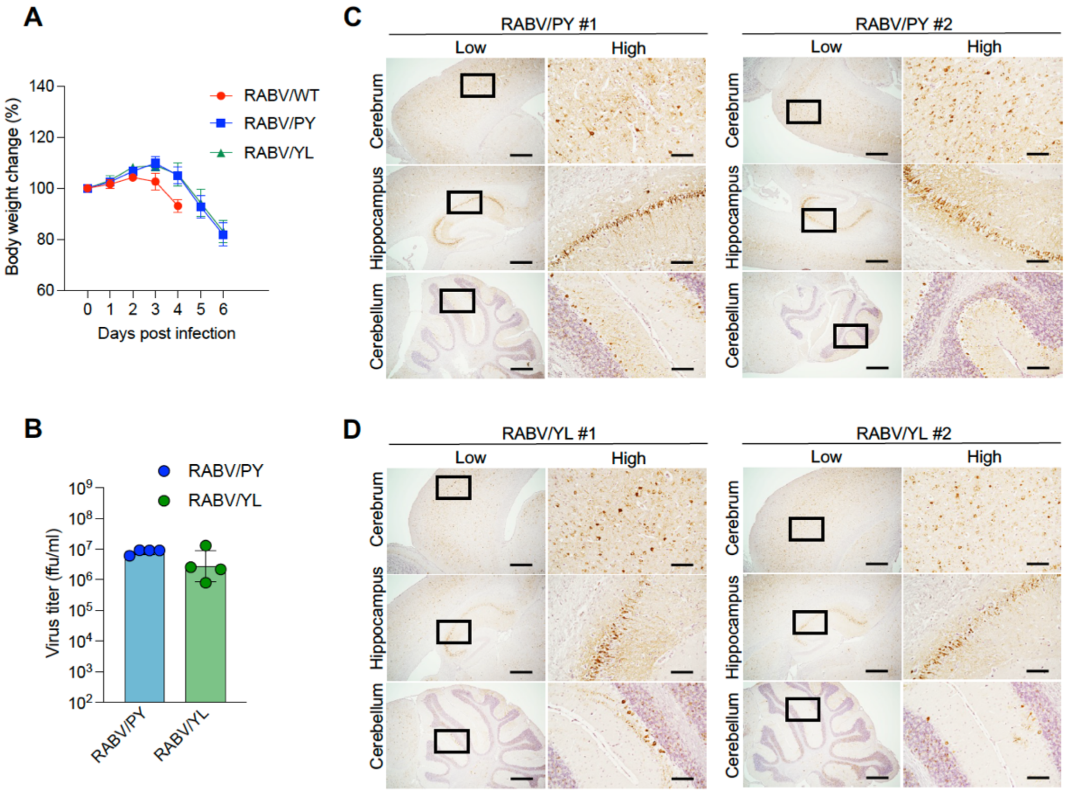

Fig. S7. Virus growth and distributions of RABV mutants in mouse brains on 6 dpi (related to Fig.6).

Five-week-old ddY mice were inoculated with 10^2 ffu RABV intracranially. Virus-infected mice were monitored for (A) body weight changes. Data in the graphs are means ± standard deviations (RABV/WT: n = 3; RABV/PY: n = 6; RABV/YL: n = 6). (B) Virus titers in brain homogenate at 6 dpi were determined using a focus forming assay. Bars show means ± standard deviations (n = 6). (C, D) Immunohistochemistry of the mouse brain. Brain sections at 6 dpi by (C) RABV/PY or (D) RABV/YL were stained with anti-RABV N. Scale bars: 500 μm (low magnification) and 100 μm (high magnification).
